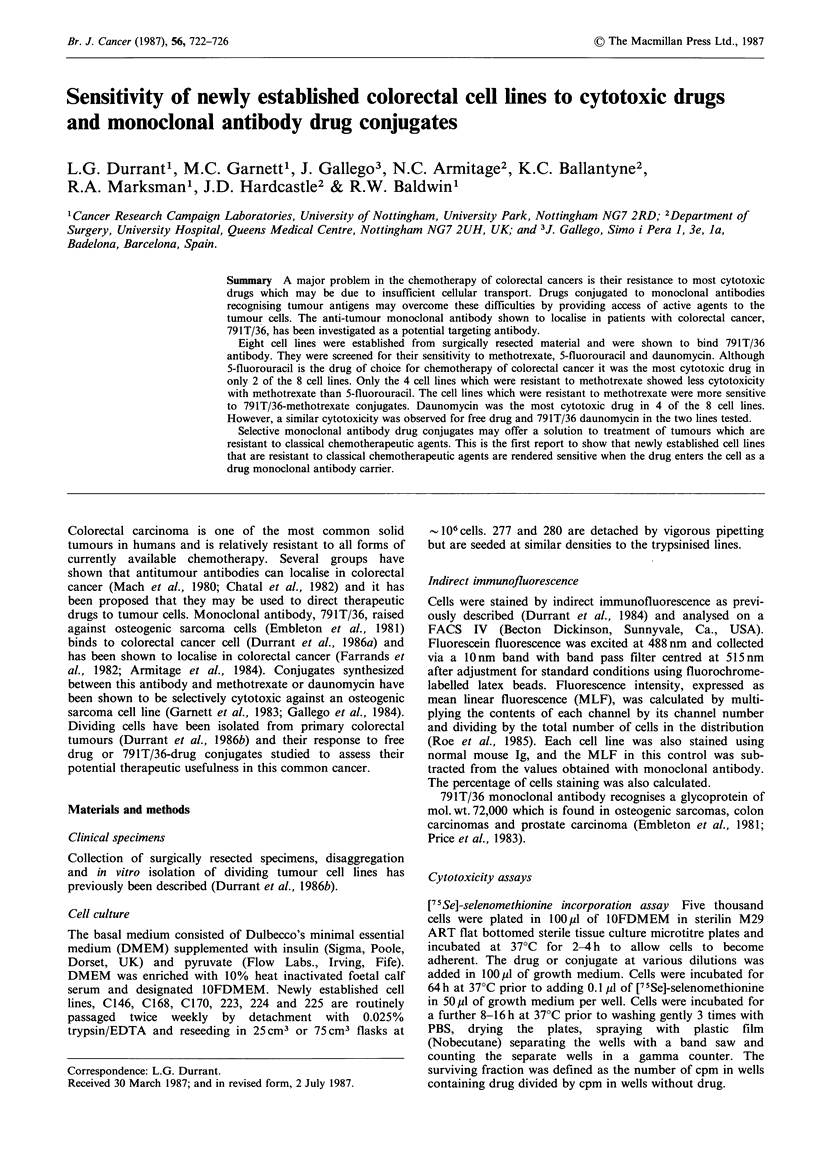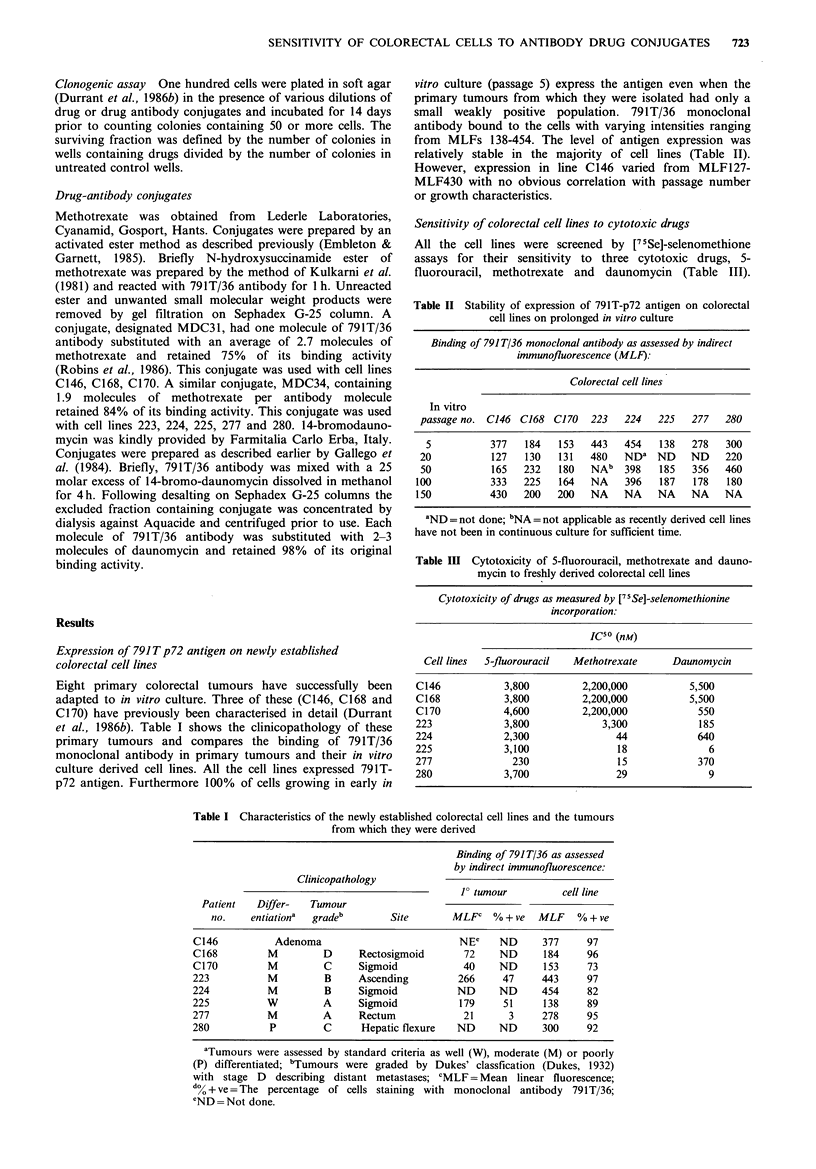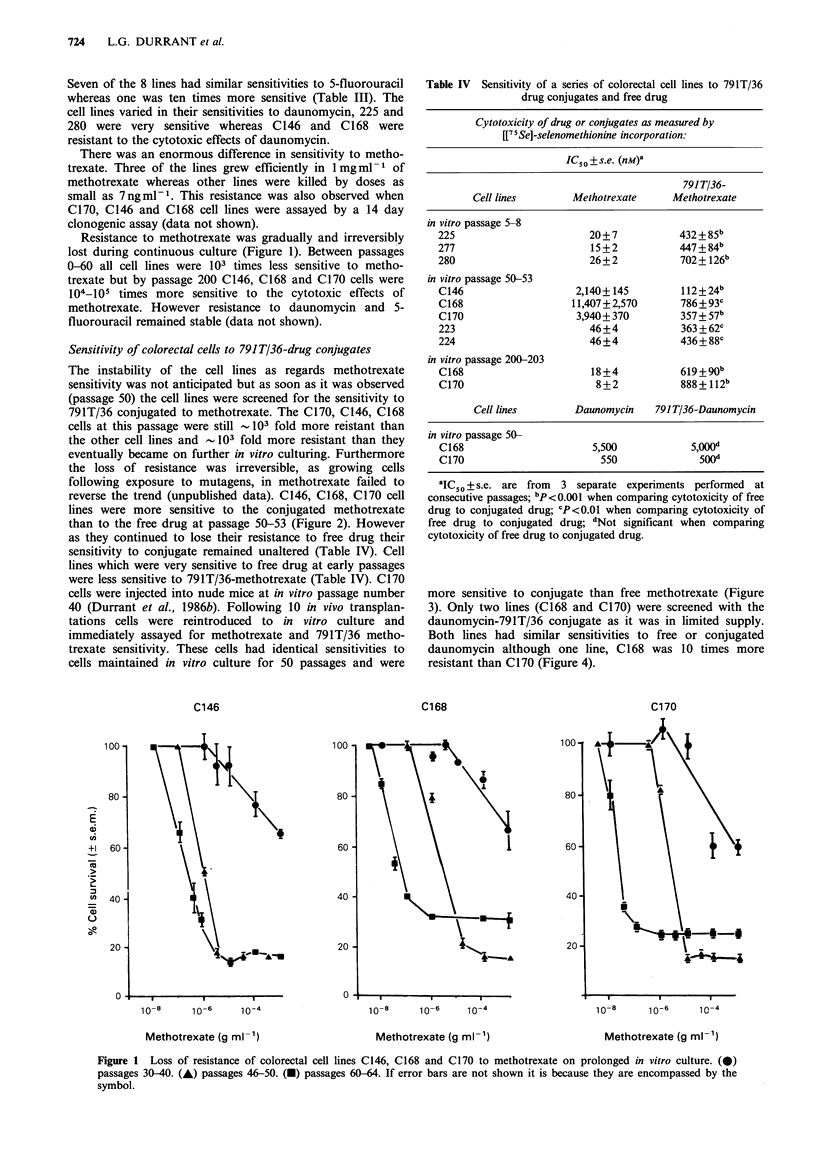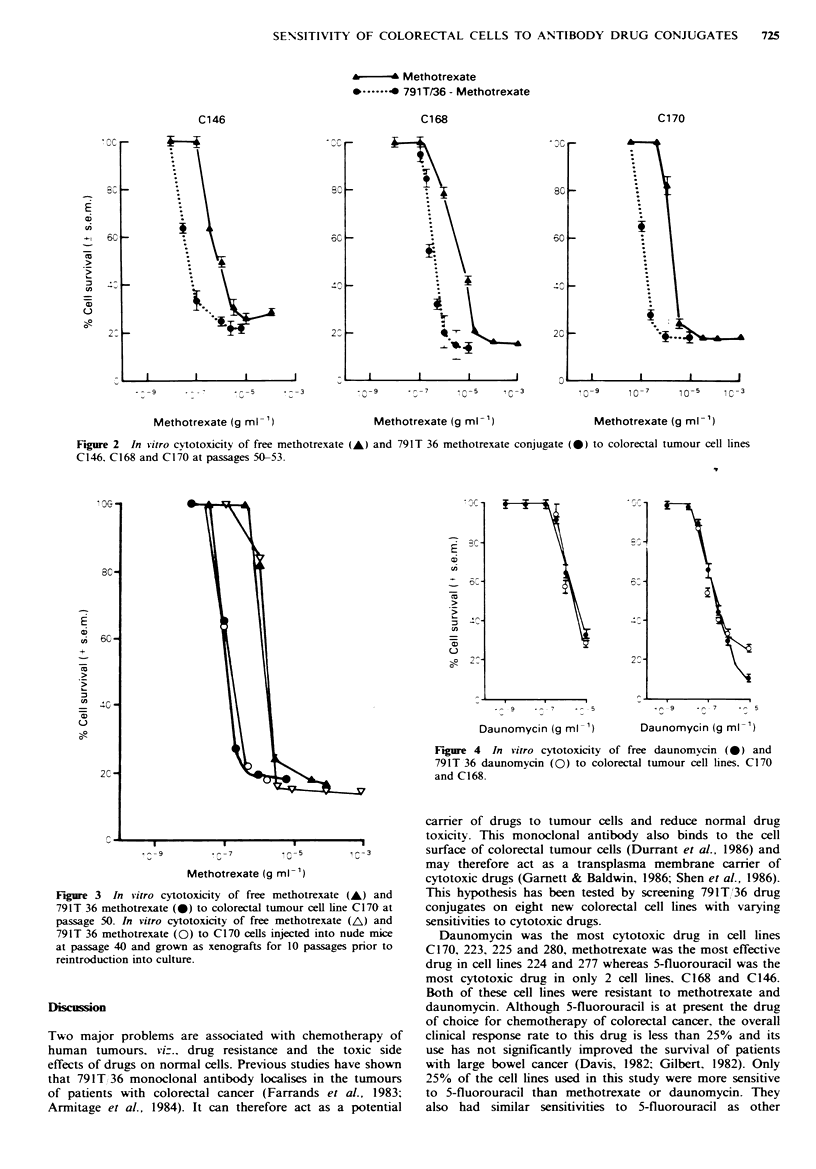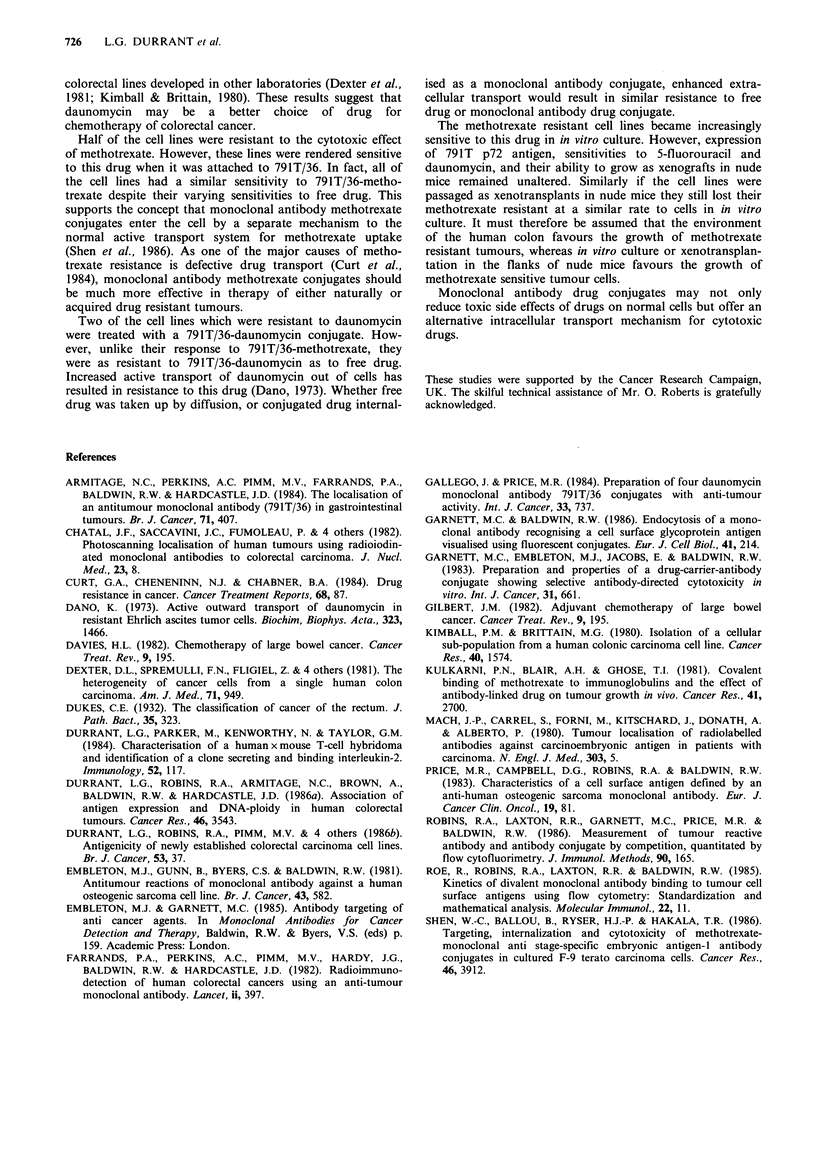# Sensitivity of newly established colorectal cell lines to cytotoxic drugs and monoclonal antibody drug conjugates.

**DOI:** 10.1038/bjc.1987.277

**Published:** 1987-12

**Authors:** L. G. Durrant, M. C. Garnett, J. Gallego, N. C. Armitage, K. C. Ballantyne, R. A. Marksman, J. D. Hardcastle, R. W. Baldwin

**Affiliations:** Cancer Research Campaign Laboratories, University of Nottingham, University Park, UK.

## Abstract

A major problem in the chemotherapy of colorectal cancers is their resistance to most cytotoxic drugs which may be due to insufficient cellular transport. Drugs conjugated to monoclonal antibodies recognising tumour antigens may overcome these difficulties by providing access of active agents to the tumour cells. The anti-tumour monoclonal antibody shown to localise in patients with colorectal cancer, 791T/36, has been investigated as a potential targeting antibody. Eight cell lines were established from surgically resected material and were shown to bind 791T/36 antibody. They were screened for their sensitivity to methotrexate, 5-fluorouracil and daunomycin. Although 5-fluorouracil is the drug of choice for chemotherapy of colorectal cancer it was the most cytotoxic drug in only 2 of the 8 cell lines. Only the 4 cell lines which were resistant to methotrexate showed less cytotoxicity with methotrexate than 5-fluorouracil. The cell lines which were resistant to methotrexate were more sensitive to 791T/36-methotrexate conjugates. Daunomycin was the most cytotoxic drug in 4 of the 8 cell lines. However, a similar cytotoxicity was observed for free drug and 791T/36 daunomycin in the two lines tested. Selective monoclonal antibody drug conjugates may offer a solution to treatment of tumours which are resistant to classical chemotherapeutic agents. This is the first report to show that newly established cell lines that are resistant to classical chemotherapeutic agents are rendered sensitive when the drug enters the cell as a drug monoclonal antibody carrier.


Br. J. Cancer (1987), 56, 722–726 © The Macmillan Press Ltd., 1987

### Sensitivity of newly established colorectal cell lines to cytotoxic drugs and monoclonal antibody drug conjugates

L.G. Durrant[1], M.C. Garnett[1], J. Gallego[3], N.C. Armitage[2], K.C. Ballantyne[2], R.A. Marksman[1], J.D. Hardcastle[2] & R.W. Baldwin[1]

[1]*Cancer Research Campaign Laboratories, University of Nottingham, University Park, Nottingham NG7 2RD;* [2]*Department of Surgery, University Hospital, Queens Medical Centre, Nottingham NG7 2UH, UK; and* [3]*J. Gallego, Simo i Pera 1, 3e, 1a, Badelona, Barcelona, Spain.*

**Summary** A major problem in the chemotherapy of colorectal cancers is their resistance to most cytotoxic drugs which may be due to insufficient cellular transport. Drugs conjugated to monoclonal antibodies recognising tumour antigens may overcome these difficulties by providing access of active agents to the tumour cells. The anti-tumour monoclonal antibody shown to localise in patients with colorectal cancer, 791T/36, has been investigated as a potential targeting antibody.

Eight cell lines were established from surgically resected material and were shown to bind 791T/36 antibody. They were screened for their sensitivity to methotrexate, 5-fluorouracil and daunomycin. Although 5-fluorouracil is the drug of choice for chemotherapy of colorectal cancer it was the most cytotoxic drug in only 2 of the 8 cell lines. Only the 4 cell lines which were resistant to methotrexate showed less cytotoxicity with methotrexate than 5-fluorouracil. The cell lines which were resistant to methotrexate were more sensitive to 791T/36-methotrexate conjugates. Daunomycin was the most cytotoxic drug in 4 of the 8 cell lines. However, a similar cytotoxicity was observed for free drug and 791T/36 daunomycin in the two lines tested.

Selective monoclonal antibody drug conjugates may offer a solution to treatment of tumours which are resistant to classical chemotherapeutic agents. This is the first report to show that newly established cell lines that are resistant to classical chemotherapeutic agents are rendered sensitive when the drug enters the cell as a drug monoclonal antibody carrier.

Colorectal carcinoma is one of the most common solid tumours in humans and is relatively resistant to all forms of currently available chemotherapy. Several groups have shown that antitumour antibodies can localise in colorectal cancer (Mach *et al.*, 1980; Chatal *et al.*, 1982) and it has been proposed that they may be used to direct therapeutic drugs to tumour cells. Monoclonal antibody, 791T/36, raised against osteogenic sarcoma cells (Embleton *et al.*, 1981) binds to colorectal cancer cell (Durrant *et al.*, 1986*a*) and has been shown to localise in colorectal cancer (Farrands *et al.*, 1982; Armitage *et al.*, 1984). Conjugates synthesized between this antibody and methotrexate or daunomycin have been shown to be selectively cytotoxic against an osteogenic sarcoma cell line (Garnett *et al.*, 1983; Gallego *et al.*, 1984). Dividing cells have been isolated from primary colorectal tumours (Durrant *et al.*, 1986*b*) and their response to free drug or 791T/36-drug conjugates studied to assess their potential therapeutic usefulness in this common cancer.

### Materials and methods

### *Clinical specimens*

Collection of surgically resected specimens, disaggregation and *in vitro* isolation of dividing tumour cell lines has previously been described (Durrant *et al.*, 1986*b*).

### *Cell culture*

The basal medium consisted of Dulbecco's minimal essential medium (DMEM) supplemented with insulin (Sigma, Poole, Dorset, UK) and pyruvate (Flow Labs., Irving, Fife). DMEM was enriched with 10% heat inactivated foetal calf serum and designated 10FDMEM. Newly established cell lines, C146, C168, C170, 223, 224 and 225 are routinely passaged twice weekly by detachment with 0.025% trypsin/EDTA and reseeding in 25 $cm^3$ or 75 $cm^3$ flasks at $\sim 10^6$ cells. 277 and 280 are detached by vigorous pipetting but are seeded at similar densities to the trypsinised lines.

### *Indirect immunofluorescence*

Cells were stained by indirect immunofluorescence as previously described (Durrant *et al.*, 1984) and analysed on a FACS IV (Becton Dickinson, Sunnyvale, Ca., USA). Fluorescein fluorescence was excited at 488 nm and collected via a 10 nm band with band pass filter centred at 515 nm after adjustment for standard conditions using fluorochrome-labelled latex beads. Fluorescence intensity, expressed as mean linear fluorescence (MLF), was calculated by multiplying the contents of each channel by its channel number and dividing by the total number of cells in the distribution (Roe *et al.*, 1985). Each cell line was also stained using normal mouse Ig, and the MLF in this control was subtracted from the values obtained with monoclonal antibody. The percentage of cells staining was also calculated.

791T/36 monoclonal antibody recognises a glycoprotein of mol. wt. 72,000 which is found in osteogenic sarcomas, colon carcinomas and prostate carcinoma (Embleton *et al.*, 1981; Price *et al.*, 1983).

### *Cytotoxicity assays*

*[$^{75}$Se]-selenomethionine incorporation assay* Five thousand cells were plated in 100 $\mu$l of 10FDMEM in sterilin M29 ART flat bottomed sterile tissue culture microtitre plates and incubated at 37°C for 2–4 h to allow cells to become adherent. The drug or conjugate at various dilutions was added in 100 $\mu$l of growth medium. Cells were incubated for 64 h at 37°C prior to adding 0.1 $\mu$l of [$^{75}$Se]-selenomethionine in 50 $\mu$l of growth medium per well. Cells were incubated for a further 8–16 h at 37°C prior to washing gently 3 times with PBS, drying the plates, spraying with plastic film (Nobecutane) separating the wells with a band saw and counting the separate wells in a gamma counter. The surviving fraction was defined as the number of cpm in wells containing drug divided by cpm in wells without drug.

Correspondence: L.G. Durrant.
Received 30 March 1987; and in revised form, 2 July 1987.

*Clonogenic assay* One hundred cells were plated in soft agar (Durrant *et al.*, 1986*b*) in the presence of various dilutions of drug or drug antibody conjugates and incubated for 14 days prior to counting colonies containing 50 or more cells. The surviving fraction was defined by the number of colonies in wells containing drugs divided by the number of colonies in untreated control wells.

*Drug-antibody conjugates*

Methotrexate was obtained from Lederle Laboratories, Cyanamid, Gosport, Hants. Conjugates were prepared by an activated ester method as described previously (Embleton & Garnett, 1985). Briefly N-hydroxysuccinamide ester of methotrexate was prepared by the method of Kulkarni *et al.* (1981) and reacted with 791T/36 antibody for 1 h. Unreacted ester and unwanted small molecular weight products were removed by gel filtration on Sephadex G-25 column. A conjugate, designated MDC31, had one molecule of 791T/36 antibody substituted with an average of 2.7 molecules of methotrexate and retained 75% of its binding activity (Robins *et al.*, 1986). This conjugate was used with cell lines C146, C168, C170. A similar conjugate, MDC34, containing 1.9 molecules of methotrexate per antibody molecule retained 84% of its binding activity. This conjugate was used with cell lines 223, 224, 225, 277 and 280. 14-bromodaunomycin was kindly provided by Farmitalia Carlo Erba, Italy. Conjugates were prepared as described earlier by Gallego *et al.* (1984). Briefly, 791T/36 antibody was mixed with a 25 molar excess of 14-bromo-daunomycin dissolved in methanol for 4 h. Following desalting on Sephadex G-25 columns the excluded fraction containing conjugate was concentrated by dialysis against Aquacide and centrifuged prior to use. Each molecule of 791T/36 antibody was substituted with 2–3 molecules of daunomycin and retained 98% of its original binding activity.

### Results

*Expression of 791T p72 antigen on newly established colorectal cell lines*

Eight primary colorectal tumours have successfully been adapted to *in vitro* culture. Three of these (C146, C168 and C170) have previously been characterised in detail (Durrant *et al.*, 1986*b*). Table I shows the clinicopathology of these primary tumours and compares the binding of 791T/36 monoclonal antibody in primary tumours and their *in vitro* culture derived cell lines. All the cell lines expressed 791T-p72 antigen. Furthermore 100% of cells growing in early *in vitro* culture (passage 5) express the antigen even when the primary tumours from which they were isolated had only a small weakly positive population. 791T/36 monoclonal antibody bound to the cells with varying intensities ranging from MLFs 138-454. The level of antigen expression was relatively stable in the majority of cell lines (Table II). However, expression in line C146 varied from MLF127-MLF430 with no obvious correlation with passage number or growth characteristics.

*Sensitivity of colorectal cell lines to cytotoxic drugs*

All the cell lines were screened by [$^{75}$Se]-selenomethione assays for their sensitivity to three cytotoxic drugs, 5-fluorouracil, methotrexate and daunomycin (Table III).

**Table II** Stability of expression of 791T-p72 antigen on colorectal cell lines on prolonged *in vitro* culture

*Binding of 791T/36 monoclonal antibody as assessed by indirect immunofluorescence (MLF):*

| In vitro passage no. | C146 | C168 | C170 | 223 | 224 | 225 | 277 | 280 |
|---|---|---|---|---|---|---|---|---|
| | *Colorectal cell lines* | | | | | | | |
| 5 | 377 | 184 | 153 | 443 | 454 | 138 | 278 | 300 |
| 20 | 127 | 130 | 131 | 480 | ND[a] | ND | ND | 220 |
| 50 | 165 | 232 | 180 | NA[b] | 398 | 185 | 356 | 460 |
| 100 | 333 | 225 | 164 | NA | 396 | 187 | 178 | 180 |
| 150 | 430 | 200 | 200 | NA | NA | NA | NA | NA |

[a]ND = not done; [b]NA = not applicable as recently derived cell lines have not been in continuous culture for sufficient time.

**Table III** Cytotoxicity of 5-fluorouracil, methotrexate and daunomycin to freshly derived colorectal cell lines

*Cytotoxicity of drugs as measured by [$^{75}$Se]-selenomethionine incorporation:*

| Cell lines | 5-fluorouracil | Methotrexate | Daunomycin |
|---|---|---|---|
| | $IC^{50}$ (nM) | | |
| C146 | 3,800 | 2,200,000 | 5,500 |
| C168 | 3,800 | 2,200,000 | 5,500 |
| C170 | 4,600 | 2,200,000 | 550 |
| 223 | 3,800 | 3,300 | 185 |
| 224 | 2,300 | 44 | 640 |
| 225 | 3,100 | 18 | 6 |
| 277 | 230 | 15 | 370 |
| 280 | 3,700 | 29 | 9 |

**Table I** Characteristics of the newly established colorectal cell lines and the tumours from which they were derived

| Patient no. | Clinicopathology: Differentiation[a] | Tumour grade[b] | Site | 1° tumour MLF[c] | 1° tumour %+ve | cell line MLF | cell line %+ve |
|---|---|---|---|---|---|---|---|
| C146 | Adenoma | | | NE[e] | ND | 377 | 97 |
| C168 | M | D | Rectosigmoid | 72 | ND | 184 | 96 |
| C170 | M | C | Sigmoid | 40 | ND | 153 | 73 |
| 223 | M | B | Ascending | 266 | 47 | 443 | 97 |
| 224 | M | B | Sigmoid | ND | ND | 454 | 82 |
| 225 | W | A | Sigmoid | 179 | 51 | 138 | 89 |
| 277 | M | A | Rectum | 21 | 3 | 278 | 95 |
| 280 | P | C | Hepatic flexure | ND | ND | 300 | 92 |

(Binding of 791T/36 as assessed by indirect immunofluorescence.)

[a]Tumours were assessed by standard criteria as well (W), moderate (M) or poorly (P) differentiated; [b]Tumours were graded by Dukes' classfication (Dukes, 1932) with stage D describing distant metastases; [c]MLF = Mean linear fluorescence; [d]% + ve = The percentage of cells staining with monoclonal antibody 791T/36; [e]ND = Not done.

Seven of the 8 lines had similar sensitivities to 5-fluorouracil whereas one was ten times more sensitive (Table III). The cell lines varied in their sensitivities to daunomycin, 225 and 280 were very sensitive whereas C146 and C168 were resistant to the cytotoxic effects of daunomycin.

There was an enormous difference in sensitivity to methotrexate. Three of the lines grew efficiently in 1 mg ml$^{-1}$ of methotrexate whereas other lines were killed by doses as small as 7 ng ml$^{-1}$. This resistance was also observed when C170, C146 and C168 cell lines were assayed by a 14 day clonogenic assay (data not shown).

Resistance to methotrexate was gradually and irreversibly lost during continuous culture (Figure 1). Between passages 0–60 all cell lines were $10^3$ times less sensitive to methotrexate but by passage 200 C146, C168 and C170 cells were $10^4$–$10^5$ times more sensitive to the cytotoxic effects of methotrexate. However resistance to daunomycin and 5-fluorouracil remained stable (data not shown).

### *Sensitivity of colorectal cells to 791T/36-drug conjugates*

The instability of the cell lines as regards methotrexate sensitivity was not anticipated but as soon as it was observed (passage 50) the cell lines were screened for the sensitivity to 791T/36 conjugated to methotrexate. The C170, C146, C168 cells at this passage were still $\sim 10^3$ fold more reistant than the other cell lines and $\sim 10^3$ fold more resistant than they eventually became on further *in vitro* culturing. Furthermore the loss of resistance was irreversible, as growing cells following exposure to mutagens, in methotrexate failed to reverse the trend (unpublished data). C146, C168, C170 cell lines were more sensitive to the conjugated methotrexate than to the free drug at passage 50–53 (Figure 2). However as they continued to lose their resistance to free drug their sensitivity to conjugate remained unaltered (Table IV). Cell lines which were very sensitive to free drug at early passages were less sensitive to 791T/36-methotrexate (Table IV). C170 cells were injected into nude mice at *in vitro* passage number 40 (Durrant *et al.*, 1986*b*). Following 10 *in vivo* transplantations cells were reintroduced to *in vitro* culture and immediately assayed for methotrexate and 791T/36 methotrexate sensitivity. These cells had identical sensitivities to cells maintained *in vitro* culture for 50 passages and were more sensitive to conjugate than free methotrexate (Figure 3). Only two lines (C168 and C170) were screened with the daunomycin-791T/36 conjugate as it was in limited supply. Both lines had similar sensitivities to free or conjugated daunomycin although one line, C168 was 10 times more resistant than C170 (Figure 4).

**Table IV** Sensitivity of a series of colorectal cell lines to 791T/36 drug conjugates and free drug

| *Cytotoxicity of drug or conjugates as measured by [[$^{75}$Se]-selenomethionine incorporation:* | | |
|---|---|---|
| | *$IC_{50}$ ± s.e. (nM)*[a] | |
| *Cell lines* | *Methotrexate* | *791T/36-Methotrexate* |
| *in vitro* passage 5–8 | | |
| 225 | 20±7 | 432±85[b] |
| 277 | 15±2 | 447±84[b] |
| 280 | 26±2 | 702±126[b] |
| *in vitro* passage 50–53 | | |
| C146 | 2,140±145 | 112±24[b] |
| C168 | 11,407±2,570 | 786±93[c] |
| C170 | 3,940±370 | 357±57[b] |
| 223 | 46±4 | 363±62[c] |
| 224 | 46±4 | 436±88[c] |
| *in vitro* passage 200–203 | | |
| C168 | 18±4 | 619±90[b] |
| C170 | 8±2 | 888±112[b] |
| *Cell lines* | *Daunomycin* | *791T/36-Daunomycin* |
| *in vitro* passage 50– | | |
| C168 | 5,500 | 5,000[d] |
| C170 | 550 | 500[d] |

[a]$IC_{50}$ ± s.e. are from 3 separate experiments performed at consecutive passages; [b]$P<0.001$ when comparing cytotoxicity of free drug to conjugated drug; [c]$P<0.01$ when comparing cytotoxicity of free drug to conjugated drug; [d]Not significant when comparing cytotoxicity of free drug to conjugated drug.

**Figure 1** Loss of resistance of colorectal cell lines C146, C168 and C170 to methotrexate on prolonged *in vitro* culture. (●) passages 30–40. (▲) passages 46–50. (■) passages 60–64. If error bars are not shown it is because they are encompassed by the symbol.

**Figure 2** *In vitro* cytotoxicity of free methotrexate (▲) and 791T 36 methotrexate conjugate (●) to colorectal tumour cell lines C146, C168 and C170 at passages 50–53.

**Figure 3** *In vitro* cytotoxicity of free methotrexate (▲) and 791T 36 methotrexate (●) to colorectal tumour cell line C170 at passage 50. *In vitro* cytotoxicity of free methotrexate (△) and 791T 36 methotrexate (○) to C170 cells injected into nude mice at passage 40 and grown as xenografts for 10 passages prior to reintroduction into culture.

**Figure 4** *In vitro* cytotoxicity of free daunomycin (●) and 791T 36 daunomycin (○) to colorectal tumour cell lines, C170 and C168.

### Discussion

Two major problems are associated with chemotherapy of human tumours, *viz.*, drug resistance and the toxic side effects of drugs on normal cells. Previous studies have shown that 791T/36 monoclonal antibody localises in the tumours of patients with colorectal cancer (Farrands *et al.*, 1983; Armitage *et al.*, 1984). It can therefore act as a potential carrier of drugs to tumour cells and reduce normal drug toxicity. This monoclonal antibody also binds to the cell surface of colorectal tumour cells (Durrant *et al.*, 1986) and may therefore act as a transplasma membrane carrier of cytotoxic drugs (Garnett & Baldwin, 1986; Shen *et al.*, 1986). This hypothesis has been tested by screening 791T/36 drug conjugates on eight new colorectal cell lines with varying sensitivities to cytotoxic drugs.

Daunomycin was the most cytotoxic drug in cell lines C170, 223, 225 and 280, methotrexate was the most effective drug in cell lines 224 and 277 whereas 5-fluorouracil was the most cytotoxic drug in only 2 cell lines, C168 and C146. Both of these cell lines were resistant to methotrexate and daunomycin. Although 5-fluorouracil is at present the drug of choice for chemotherapy of colorectal cancer, the overall clinical response rate to this drug is less than 25% and its use has not significantly improved the survival of patients with large bowel cancer (Davis, 1982; Gilbert, 1982). Only 25% of the cell lines used in this study were more sensitive to 5-fluorouracil than methotrexate or daunomycin. They also had similar sensitivities to 5-fluorouracil as other colorectal lines developed in other laboratories (Dexter et al., 1981; Kimball & Brittain, 1980). These results suggest that daunomycin may be a better choice of drug for chemotherapy of colorectal cancer.

Half of the cell lines were resistant to the cytotoxic effect of methotrexate. However, these lines were rendered sensitive to this drug when it was attached to 791T/36. In fact, all of the cell lines had a similar sensitivity to 791T/36-methotrexate despite their varying sensitivities to free drug. This supports the concept that monoclonal antibody methotrexate conjugates enter the cell by a separate mechanism to the normal active transport system for methotrexate uptake (Shen et al., 1986). As one of the major causes of methotrexate resistance is defective drug transport (Curt et al., 1984), monoclonal antibody methotrexate conjugates should be much more effective in therapy of either naturally or acquired drug resistant tumours.

Two of the cell lines which were resistant to daunomycin were treated with a 791T/36-daunomycin conjugate. However, unlike their response to 791T/36-methotrexate, they were as resistant to 791T/36-daunomycin as to free drug. Increased active transport of daunomycin out of cells has resulted in resistance to this drug (Dano, 1973). Whether free drug was taken up by diffusion, or conjugated drug internalised as a monoclonal antibody conjugate, enhanced extracellular transport would result in similar resistance to free drug or monoclonal antibody drug conjugate.

The methotrexate resistant cell lines became increasingly sensitive to this drug in *in vitro* culture. However, expression of 791T p72 antigen, sensitivities to 5-fluorouracil and daunomycin, and their ability to grow as xenografts in nude mice remained unaltered. Similarly if the cell lines were passaged as xenotransplants in nude mice they still lost their methotrexate resistant at a similar rate to cells in *in vitro* culture. It must therefore be assumed that the environment of the human colon favours the growth of methotrexate resistant tumours, whereas *in vitro* culture or xenotransplantation in the flanks of nude mice favours the growth of methotrexate sensitive tumour cells.

Monoclonal antibody drug conjugates may not only reduce toxic side effects of drugs on normal cells but offer an alternative intracellular transport mechanism for cytotoxic drugs.

These studies were supported by the Cancer Research Campaign, UK. The skilful technical assistance of Mr. O. Roberts is gratefully acknowledged.

**References**

ARMITAGE, N.C., PERKINS, A.C. PIMM, M.V., FARRANDS, P.A., BALDWIN, R.W. & HARDCASTLE, J.D. (1984). The localisation of an antitumour monoclonal antibody (791T/36) in gastrointestinal tumours. *Br. J. Cancer*, **71**, 407.

CHATAL, J.F., SACCAVINI, J.C., FUMOLEAU, P. & 4 others (1982). Photoscanning localisation of human tumours using radioiodinated monoclonal antibodies to colorectal carcinoma. *J. Nucl. Med.*, **23**, 8.

CURT, G.A., CHENENINN, N.J. & CHABNER, B.A. (1984). Drug resistance in cancer. *Cancer Treatment Reports*, **68**, 87.

DANO, K. (1973). Active outward transport of daunomycin in resistant Ehrlich ascites tumor cells. *Biochim, Biophys. Acta.*, **323**, 1466.

DAVIES, H.L. (1982). Chemotherapy of large bowel cancer. *Cancer Treat. Rev.*, **9**, 195.

DEXTER, D.L., SPREMULLI, F.N., FLIGIEL, Z. & 4 others (1981). The heterogeneity of cancer cells from a single human colon carcinoma. *Am. J. Med.*, **71**, 949.

DUKES, C.E. (1932). The classification of cancer of the rectum. *J. Path. Bact.*, **35**, 323.

DURRANT, L.G., PARKER, M., KENWORTHY, N. & TAYLOR, G.M. (1984). Characterisation of a human × mouse T-cell hybridoma and identification of a clone secreting and binding interleukin-2. *Immunology*, **52**, 117.

DURRANT, L.G., ROBINS, R.A., ARMITAGE, N.C., BROWN, A., BALDWIN, R.W. & HARDCASTLE, J.D. (1986*a*). Association of antigen expression and DNA-ploidy in human colorectal tumours. *Cancer Res.*, **46**, 3543.

DURRANT, L.G., ROBINS, R.A., PIMM, M.V. & 4 others (1986*b*). Antigenicity of newly established colorectal carcinoma cell lines. *Br. J. Cancer*, **53**, 37.

EMBLETON, M.J., GUNN, B., BYERS, C.S. & BALDWIN, R.W. (1981). Antitumour reactions of monoclonal antibody against a human osteogenic sarcoma cell line. *Br. J. Cancer*, **43**, 582.

EMBLETON, M.J. & GARNETT, M.C. (1985). Antibody targeting of anti cancer agents. In *Monoclonal Antibodies for Cancer Detection and Therapy*, Baldwin, R.W. & Byers, V.S. (eds) p. 159. Academic Press: London.

FARRANDS, P.A., PERKINS, A.C., PIMM, M.V., HARDY, J.G., BALDWIN, R.W. & HARDCASTLE, J.D. (1982). Radioimmunodetection of human colorectal cancers using an anti-tumour monoclonal antibody. *Lancet*, **ii**, 397.

GALLEGO, J. & PRICE, M.R. (1984). Preparation of four daunomycin monoclonal antibody 791T/36 conjugates with anti-tumour activity. *Int. J. Cancer*, **33**, 737.

GARNETT, M.C. & BALDWIN, R.W. (1986). Endocytosis of a monoclonal antibody recognising a cell surface glycoprotein antigen visualised using fluorescent conjugates. *Eur. J. Cell Biol.*, **41**, 214.

GARNETT, M.C., EMBLETON, M.J., JACOBS, E. & BALDWIN, R.W. (1983). Preparation and properties of a drug-carrier-antibody conjugate showing selective antibody-directed cytotoxicity *in vitro*. *Int. J. Cancer*, **31**, 661.

GILBERT, J.M. (1982). Adjuvant chemotherapy of large bowel cancer. *Cancer Treat. Rev.*, **9**, 195.

KIMBALL, P.M. & BRITTAIN, M.G. (1980). Isolation of a cellular sub-population from a human colonic carcinoma cell line. *Cancer Res.*, **40**, 1574.

KULKARNI, P.N., BLAIR, A.H. & GHOSE, T.I. (1981). Covalent binding of methotrexate to immunoglobulins and the effect of antibody-linked drug on tumour growth *in vivo*. *Cancer Res.*, **41**, 2700.

MACH, J.-P., CARREL, S., FORNI, M., KITSCHARD, J., DONATH, A. & ALBERTO, P. (1980). Tumour localisation of radiolabelled antibodies against carcinoembryonic antigen in patients with carcinoma. *N. Engl. J. Med.*, **303**, 5.

PRICE, M.R., CAMPBELL, D.G., ROBINS, R.A. & BALDWIN, R.W. (1983). Characteristics of a cell surface antigen defined by an anti-human osteogenic sarcoma monoclonal antibody. *Eur. J. Cancer Clin. Oncol.*, **19**, 81.

ROBINS, R.A., LAXTON, R.R., GARNETT, M.C., PRICE, M.R. & BALDWIN, R.W. (1986). Measurement of tumour reactive antibody and antibody conjugate by competition, quantitated by flow cytofluorimetry. *J. Immunol. Methods*, **90**, 165.

ROE, R., ROBINS, R.A., LAXTON, R.R. & BALDWIN, R.W. (1985). Kinetics of divalent monoclonal antibody binding to tumour cell surface antigens using flow cytometry: Standardization and mathematical analysis. *Molecular Immunol.*, **22**, 11.

SHEN, W.-C., BALLOU, B., RYSER, H.J.-P. & HAKALA, T.R. (1986). Targeting, internalization and cytotoxicity of methotrexate-monoclonal anti stage-specific embryonic antigen-1 antibody conjugates in cultured F-9 terato carcinoma cells. *Cancer Res.*, **46**, 3912.